# Insight on Oxygen-Supplying Biomaterials Used to Enhance Cell Survival, Retention, and Engraftment for Tissue Repair

**DOI:** 10.3390/biomedicines11061592

**Published:** 2023-05-30

**Authors:** Muhammad Rafique, Onaza Ali, Muhammad Shafiq, Minghua Yao, Kai Wang, Hiroyuki Ijima, Deling Kong, Masato Ikeda

**Affiliations:** 1 Key Laboratory of Bioactive Materials (Ministry of Education), State Key Laboratory of Medicinal Chemical Biology, College of Life Sciences, Nankai University, Tianjin 300071, China; 2 United Graduate School of Drug Discovery and Medical Information Sciences, Gifu University, 1-1 Yanagido, Gifu 501-1193, Japan; 3 Department of Chemical Engineering, Faculty of Engineering, Graduate School, Kyushu University, Fukuoka 819-0395, Japan; 4 Department of Ultrasound, Shanghai General Hospital, School of Medicine, Shanghai Jiao Tong University, Shanghai 201620, China; 5 Institute of Nano-Life-Systems, Institutes of Innovation for Future Society, Nagoya University, Furo-cho, Chikusa-ku, Nagoya 464-8603, Japan; 6 Institute for Glyco-Core Research (iGCORE), Gifu University, 1-1 Yanagido, Gifu 501-1193, Japan

**Keywords:** cell spheroids, hard tissues, oxygen carriers, oxygen-generating biomaterials, regenerative medicine, soft tissues, tissue engineering

## Abstract

Oxygen is one of the essential requirements for cell survival, retention, and proliferation. The field of regenerative medicine and tissue engineering (TE) has realized considerable achievements for the regeneration of tissues. However, tissue regeneration still lacks the full functionality of solid organ implantations; limited cell survival and retention due to oxidative stress and hypoxia in the deeper parts of tissues remains a perpetual challenge. Especially prior to neovascularization, hypoxia is a major limiting factor, since oxygen delivery becomes crucial for cell survival throughout the tissue-engineered construct. Oxygen diffusion is generally limited in the range 100–200 μm of the thickness of a scaffold, and the cells located beyond this distance face oxygen deprivation, which ultimately leads to hypoxia. Furthermore, before achieving functional anastomosis, implanted tissues will be depleted of oxygen, resulting in hypoxia (<5% dissolved oxygen) followed by anoxic (<0.5% dissolved oxygen) microenvironments. Different types of approaches have been adopted to establish a sustained oxygen supply both in vitro and in vivo. In this review, we have summarized the recent developments in oxygen-generating and/or releasing biomaterials for enhancing cell survival in vitro, as well as for promoting soft and hard tissue repair, including skin, heart, nerve, pancreas, muscle, and bone tissues in vivo. In addition, redox-scavenging biomaterials and oxygenated scaffolds have also been highlighted. The surveyed results have shown significant promise in oxygen-producing biomaterials and oxygen carriers for enhancing cell functionality for regenerative medicine and TE applications. Taken together, this review provides a detailed overview of newer approaches and technologies for oxygen production, as well as their applications for bio-related disciplines.

## 1. Introduction

The rise in world population, especially ageing individuals, has increased the likelihood of impaired tissues or organs owing to multiple factors. Organ transplantation remains a crucial requirement for the survival of affected individuals. Consequently, different types of organs, including kidneys, livers, hearts, bone marrow (BM), retinas, blood vessels, etc. have been successfully transplanted worldwide. Nevertheless, organ transplantation is hampered by several limitations, including the shortage of an appropriate number of donors, inefficiency of transplantable organs owing to prior deficiencies, high cost, and need for the perpetual immunosuppression. The development of biocompatible oxygen carriers (OCs) and several biomedical targeted scaffolds or devices for tissue engineering (TE) and regenerative medicine applications has remained a long-term goal for an optimal integration of biomaterials and subsequent tissue repair within the implanted biomaterials [1,2]. A previous report comprehensively demonstrated the available oxygen delivery approaches that included hyperbaric oxygen therapy (HBO), perfusion- or bioreactor-based approaches, prevascularization, and oxygen-releasing materials, as well as alternative O_2_-producing biomaterials based on the conversion of oxygen-releasing materials into oxygen vectors. The study also extensively demonstrated the effects of oxygen delivery on cell survival in vitro, as well as oxygenated scaffolds for bone and skin models in vivo. Meanwhile, in the current report we demonstrate recent developments in oxygenated scaffolds/biomaterials for enhancing cell survival and viability under hypoxic conditions in vitro, as well as the repair of multiple soft and hard body tissues, including skin, heart, nerve, pancreas, muscle, and bone tissues. Additionally, we have introduced approaches to simultaneously scavenge reactive oxygen species (ROS) and furnish oxygen, as well as mitigate hypoxia in tissue-engineered constructs and injured tissues and tumors. It is noteworthy that previous reports have mainly focused on OCs for enhancing cell survival and retention in vitro, and only a few studies have comprehensively outlined the applications of oxygen-producing biomaterials for cell survival, retention, and engraftment for both in vitro and in vivo applications [3].

Biocompatible and biodegradable scaffolds offer an alternative paradigm for regenerative medicine and TE; they are generally fabricated by using cells, scaffolds, and micro/nanofabrication techniques to create tissue-engineered constructs. These tissue-engineered constructs have already shown significant potential for multiple bio-related disciplines, such as in vitro models for drug testing and toxicity screening and in vivo surrogates for impaired tissues and organs. However, the lack of in vivo-like microenvironments and factors including extracellular matrix (ECM) composition and localization, soluble and tethered growth factors (GFs), vascular networks for the diffusion and transport of oxygen and nutrients, and ECM-like mechano-sensitivity impede the clinical translation of these tissue-engineered constructs. Of note, poor diffusion of oxygen and transport of nutrients may pose apoptosis and anoikis risks to these engineered constructs, as well as risks of poor survival and engraftment at the targeted tissues and organs after transplantation. Constructs with a thickness of only a few hundred microns face acute shortage of oxygen and hypoxic-like conditions, particularly in the deep regions of the constructs. This is further exacerbated in cell types which require more oxygen demand for their metabolic activities, such as neurons, hepatocytes, and cardiomyocytes (CMs). Therefore, a sufficient and timely supply of oxygen may potentially improve cell survival, retention, and engraftment in vitro and in vivo [4].

**Scheme 1:**
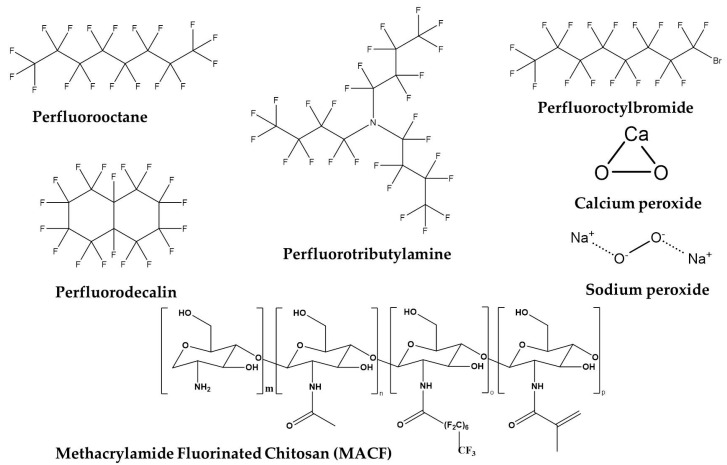
Chemical structures of some oxygen-generating materials.

So far, different types of oxygen-releasing biomaterials have been evaluated to overcome hypoxia-mediated cell loss, as well as improve tissue perfusion in in vivo conditions (Scheme 1). These include solid peroxides (i.e., calcium peroxide, CaO_2_), sodium percarbonates (Na_2_H_3_CO_6_), urea peroxide, polymer-based OCs, hemoglobin and myoglobin substitutes, hydrogen peroxide (H_2_O_2_), perfluorodecalin-encapsulated albumin nanoparticles (NPs), and perfluorocarbons (PFCs). A solution of native hemoglobin shows sigmoidal O_2_ dissociation behavior, which is similar to the response of whole blood. The most widely used solid peroxides include CaO_2_ and magnesium peroxide (MgO_2_). The mechanism of oxygen release from CaO_2_ and MgO_2_ is based on their dissociation into their respective hydroxides and H_2_O_2_ after reaction with water; H_2_O_2_ further splits into water and singlet oxygen. Similarly, after reaction with water, sodium percarbonates dissociate into sodium and carbonate ions along with H_2_O_2._ Furthermore, H_2_O_2_ can also be employed as an oxygen carrier. However, the major drawbacks of both peroxides and percarbonates, as well as H_2_O_2,_ may include an overproduction of ROS [3]. PFCs are composed of fluorinated carbon chains with complete fluorination of the carbon skeleton; these chains are more biocompatible and can dissolve a sufficient amount of oxygen due to the high electronegativity of fluorine. PFCs are chemically inert, commercially available, and can be easily sterilized for use as synthetic OCs [5]. PFCs can dissolve oxygen and release it into the cell culture environment to prolong cell growth and viability in hypoxic conditions [6]. PFCs show a linear relationship between oxygen partial pressure (*P*O_2_) and concentration. At standard temperature and pressure, the solubility of oxygen in water is 2.2 mM. This value can be much higher (e.g., 44 mM for PFCs, thereby representing a 20-fold increase over oxygen solubility in water).

**Figure 1:**
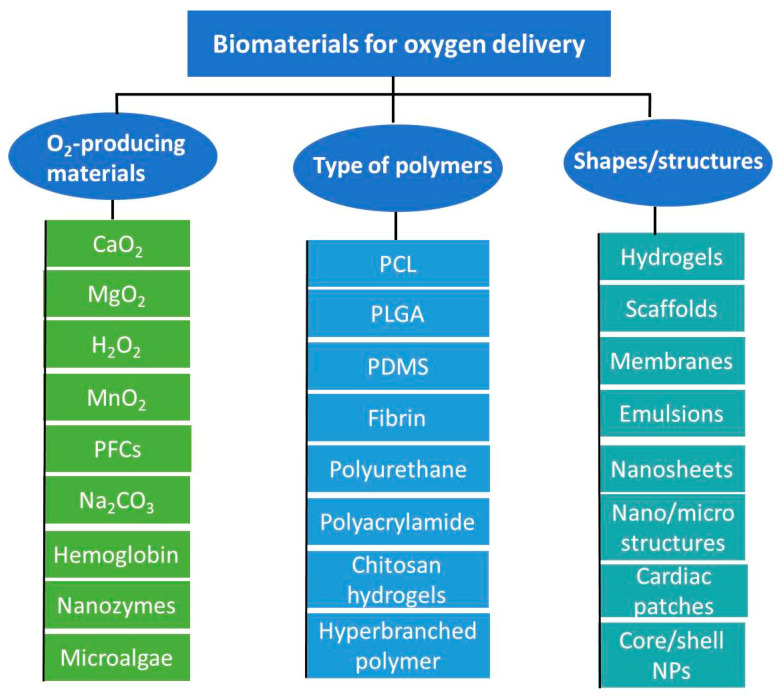
A flow chart demonstrating the types of oxygen carriers being employed, a variety of polymers, and their different shapes/types/structures as oxygen-delivery scaffolds. Calcium peroxide, CaO_2_; magnesium peroxide, MgO_2_; hydrogen peroxide, H_2_O_2_; manganese oxide, MnO_2_; perfluorocarbons, PFCs; sodium carbonate, Na_2_CO_3_; polycaprolactone, PCL; poly(L-lactide-co-glycolide), PLGA; polydimethylsiloxane, PDMS; nanoparticles, NPs.

Consequently, to overcome the aforementioned limitations, different types of OCs and biomaterials with varying shapes and structures have been exploited (Figure 1). Most of the previous reports have mainly focused on the classification of oxygen-producing reagents, synthesis and mechanisms of action, application of oxygen delivery for cell culture in vitro, and the fabrication of spheroids and tissue-engineered constructs in vitro. Recently, several reports have enumerated the beneficial effect of oxygen-producing scaffolds for in situ tissue regeneration. Consequently, herein, we not only surveyed the application of oxygen-producing biomaterials for in vitro cell manipulations (e.g., cell culture, cell spheroids, and tissue-engineered constructs) but also comprehensively analyzed the beneficial effects of oxygen-producing scaffolds for the regeneration of different types of tissues in vivo. We have discussed oxygen-producing biomaterials for enhancing cell survival, retention, and engraftment in vitro, as well as for the regeneration of different types of soft and hard body tissues (e.g., skin, heart, nerve, pancreas, muscle, and bone tissues). Moreover, we have also explored the role of oxygen delivery for scavenging ROS, mitigating tumor hypoxia, and enhancing the effectiveness of cancer therapies, including photodynamic therapy. We have subdivided our review into three different sections by mainly focusing on: (i) cells, spheroids, and organoids, (ii) soft tissues, those including skin, heart, nerve, pancreas, and muscle, and (iii) hard tissues, including bone. The most commonly used OCs, along with several types of polymers employed in specific shapes or structures, are shown in Figure 1.

## 2. Oxygenated Scaffolds to Enhance Cell Survival In Vitro

As mentioned above, for large-sized tissue-engineered constructs, cell survival depends upon the sufficient diffusion of oxygen and the transport of nutrients from adjacent blood vessels and tissues; however, this is limited to a 100–200 μm distance from the adjacent blood vessels. Cells and tissues located beyond this threshold distance may not receive an oxygen supply and may undergo necrosis and apoptosis. Oxygen-generating biomaterials (OGBs) may permit a reductionist approach to promote cell survival and promote neovascularization (Table 1). Oh et al. fabricated poly(D,L-lactide-co-glycolide) (PLGA)-based three-dimensional (3D) scaffolds and incorporated CaO_2_-based particles as OCs. The viability of 3T3 fibroblasts was assessed under normoxic and hypoxic conditions in vitro. The scaffolds ensured a continuous oxygen production for up to 10 d in vitro and remarkably improved cell viability and neo-tissue formation compared to the control groups [7].

Medium-sized scaffolds may receive sufficient oxygen supply from nearby tissues and vessels, whereas large-sized implants may not achieve an appropriate level of oxygen, especially during the prevascular phase. Farzin et al. blended CaO_2_ with polycaprolactone (PCL); the oxygenated scaffolds showed sustained and controlled release of oxygen, as well as the production of vascular endothelial growth factor (VEGF). The hydrophobic oxygen generators based on CaO_2_ promoted cell survival under hypoxic conditions and enhanced the production of VEGF compared to the control groups. The oxygen delivery further suppressed the production of H_2_O_2_ and improved cell survival [8]. It is noteworthy that the amount of the released oxygen, as well as its release rate, is very important to clearly delineate the effect of oxygen-producing biomaterials for regulating cellular and tissue repair processes. The rate of oxygenation depends on various factors, such as pH, temperature, release of oxygen-releasing materials, diffusion of water inside the scaffold, and exposure to an aqueous environment. A burst release of oxygen can lead to oxidative damage to the cells. Oxygen release can be measured by using a dissolved oxygen meter, blood gas analyzer, oxygen sensor, or an oxygen probe. The amount of released O_2_ can be controlled by manipulating different types of parameters, such as temperature (T), pH, the purity of peroxides, and their decomposition rate. CaO_2_ has been shown to generate higher oxygen production at an elevated temperature than at a low temperature [9,10].

Motealleh et al. fabricated PFC-based O_2_-carrying nanomaterials (PMOF) and incorporated them into sodium alginate (Algl) to produce Algl-PMOF-based bio-inks. These bio-inks generated 3D-printed injectable hydrogel scaffolds. O_2_ was released for up to 14 days. PMOF and AlgL-PMOF significantly improved the viability of fibroblasts under normoxic and hypoxic conditions. In contrast, these injectable scaffolds inhibited the survival of cancerous cells, including Colo818 and immortal cells under hypoxic conditions. Intriguingly, the application of oxygen-producing scaffolds along with doxycycline (DOX) reduced the viability of cancer cells, while it improved the survival of healthy cells. Therefore, the local release of O_2_ may be beneficial to improve the effectiveness of anticancer drugs for cancer therapy [11].

Different types of peroxides, including CaO_2_, MgO_2_, sodium peroxide (Na_2_O_2_), and H_2_O_2,_ have been exploited as oxygen sources. Liu et al. fabricated CaO_2_-encapsulated PLGA microspheres which were further suspended into a hydrogel system to obtain a 3D oxygen release system. The hydrogel acted as a carrier, protected the microspheres, and favored long-term oxygen release in a continuous and sustained manner. The 3D scaffolds provided the sustained release of O_2_ (ca. 45 mmHg) for up to 21 days, as compared to the control group, which exhibited oxygen release for only up to 3 days. The 3D scaffolds were also found to be superior in enhancing the survival and growth of mesenchymal stem cells (MSCs) for up to 28 days under hypoxic conditions [12].

**Figure 2:**
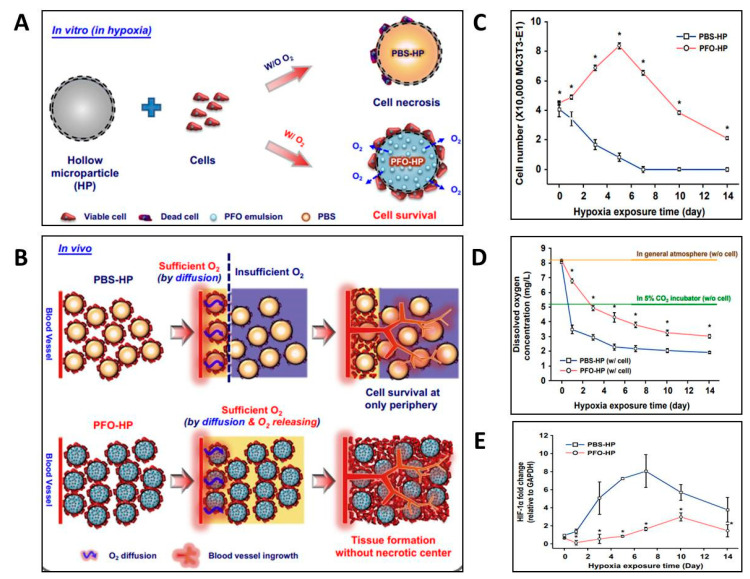
Effect of oxygen release from perfluorooctane (PFO)-based hollow MPs on cell survival and 3D tissue formation. (**A**) Schematic for in vitro cell survival in the absence or presence of PFO-HPs (PCL-based HPs loaded with PFO emulsion) and (**B**) in vivo oxygen diffusion promoting tissue formation. (**C**) Survival of MC3T3 cells under hypoxia in the presence of phosphate-buffered saline (PBS)-HP or PFO-HPs (*n* = 3; * *p* < 0.05). (**D**) Concentration of dissolved oxygen in the culture medium. (**E**) Expression of hypoxia-inducible factor 1 alpha (HIF-1α) in MC3T3 cells cultured along with PBS-HP or PFO-HPs. (*n* = 3; * *p* < 0.05). The figure was adopted from ref [14] with permission from Elsevier, Limited.

Forget et al. investigated a new approach based on oxygen-releasing microparticles (MPs). Briefly, a polydimethylsiloxane (PDMS)-based substrate was oxidized and filled with CaO_2_ or urea peroxide-based MPs. The oxygen-releasing peroxides were sandwiched between a base layer and an outer layer of plasma polymer films. The oxygen-coated surface-bearing peroxides were able to release free oxygen in the aqueous environment. Urea peroxide-based coatings were found to be more effective at enhancing the survival of MIN6 cells under hypoxic conditions [13]. Lee et al. developed PCL-based holoparticles (HPs) and loaded perfluorooctane emulsion into them to create PFO-HPs (Figure 2). The PFO-HPs increased MC3T3-E1 cells’ survival under hypoxic conditions for up to 10 days in vitro, which was significantly higher than the control group as evident in Figure 2A–C. The improved survival of cells is attributable to the higher oxygen availability from the microporous structure of HPs, which significantly promoted the survival of attached cells throughout the scaffold.

While the oxygen concentration was gradually reduced in both groups, the dissolved oxygen remained greater (5 mg/L) at day 3 and (3.2 mg/L) at day 10 in the PFO-HPs group, while it was measured at only 3.4 mg/L in the control group at day 1 (Figure 2D). In addition, the cellular responses in terms of the expression of hypoxia markers, such as hypoxia-inducible factor 1 alpha (HIF-1α) genes, were reduced owing to the availability of a continuous oxygen supply provided by PFO in the surrounding environment, as shown in Figure 2E. Thus, PFO-HPs afforded the continuous availability of oxygen due to their dense structure and even caused reduced cell necrosis even in low-oxygen-availability regions [14]. As evident from data shown in Figure 2, these HPs promoted cell survival, not only at the periphery, but also in the inner regions of the scaffolds through improved oxygen supply, which enhanced cell numbers and avoided hypoxia by elevating the dissolved oxygen concentration.

Nano-sized OCs have also been developed and used as oxygen delivery systems towards targeted sites [15,16]. However, the residual surfactants, as well as internalization of OCs through endocytosis, may pose biocompatibility concerns, thereby compromising the adaptability of this approach. Inspired by the natural biconcave shape of human red blood cells (hRBCs), Fu et al. fabricated micron-sized OCs. The core/shell type OCs were assembled by using shirasu porous glass (SPG)-based technology. The core was composed of perfluorooctyl bromide (PFOB), while the shell was comprised of mechano-elastic and biodegradable poly(lactide-co-ε-caprolactone) (PLCL) copolymers. The fabricated OCs were structurally deformable and passed through a blood capillary model, which favored the survival of HeLa cells under hypoxic conditions through continuous delivery of oxygen at sub-cellular levels [17].

**Table 1:** Oxygenated scaffolds to enhance cell survival in vitro.

Material Type	Oxygen Carrier	Beneficial Effects	Ref.
Poly(L-lactide-co-glycolic acid) (PLGA)	Calcium peroxide (CaO_2_)	O_2_ release for 10 d Cell viability increased New tissue formation vs. control group increased	[7]
Polycaprolactone (PCL)	CaO_2_	Long term oxygenation VEGF production increased H_2_O_2_ production decreased Cell survival increased	[8]
Alginate-based hydrogel	Periodic mesoporous organosilica (PMO) + perfluorocarbon (PFC)	O_2_ released and viability of fibroblasts increased Survival of tumor cells decreased	[11]
Oligopeptide-based hydrogel	PLGA/CaO_2_-loaded microsphere	O_2_ released for up to 21 days vs. control group Survival of mesenchymal stem cells (MSCs) increased	[12]
PCL hollow MPs loaded with PFO emulsion	Perfluorooctane (PFO)	Cell survival increased under hypoxia for up to 10 d Survival of MC3T3-E1 cells increased for up to 14 d	[14]
Micro-sized OCs inspired by human red blood cells (RBCs)	Perfluorooctyl bromide (PFOB) Inner core fabricated from PFOB and shell prepared from PLCL	Survival of HeLa cells increased	[17]

Cell transplantation is being intensively studied for the treatment of ischemic diseases. However, most of the transplanted cells are usually lost during in vitro cell manipulations and in vivo implantation presumably due to poor cell–cell and cell–ECM interactions as well as an oxidative microenvironment at the injury site, which leads to poor cell survival, retention, and engraftment [18]. In an in vivo microenvironment, soluble and tethered ligands, as well as ECM composition and localization, provide ubiquitous cues for cell–cell and cell–ECM interactions. During in vitro cell culture, in the absence of cell instructive cues, cells undergo apoptosis and anoikis owing to the lack of in vivo-like cell–cell and cell–ECM interactions. Moreover, poor diffusion and transport of oxygen, especially for thick tissue-engineered constructs and spheroids may further compromise cell survival. Appropriate carriers and cell-instructive biomaterials manifesting bioactive cues, as well as suitable porosity and pore size, may help overcome the abovementioned limitations. Moreover, carriers capable of furnishing the sustained release of oxygen may help overcome hypoxic effects in spheroids and thick tissue-engineered constructs.

While a series of carriers has been developed that manifest oxygen preservation strategies, they often lack gelation at 37 °C, which limits their cellular delivery into ischemic tissues. *N*-isopropylacrylamide (NIPAAm)-based hydrogels exhibit sol-to-gel transition in the physiological temperature range and have been widely exploited to harvest cell sheets in vitro. Niu et al. conjugated PFCs with NIPAAm and leveraged them to improve the survival and retention of MSCs and adipose-derived stem cells (ADSCs) in hypoxic conditions. Briefly, the hydrogels were synthesized by reversible addition–fragmentation chain reaction (RAFT) polymerization of NIPPAm, acrylate-oligolactide, 2-hydroxyethyl methacrylate, and methacrylate-polyethylene glycol-perfluorooctane. It is worthy to note that PFCs possess distinct oxygen solubility. Owing to the in situ gelation of NIPAAm-PFC conjugates, they significantly promoted cell survival, retention, and engraftment. These hydrogels exhibited higher pO_2_ than that of PBS, and promoted cell survivability under hypoxic conditions (<1% O_2_) in vitro [19].

### 2.1. Oxygenated Scaffolds to Enhance Soft Tissue Regeneration

#### 2.1.1. Skin Repair

While short-term hypoxia improves the production of angiogenic GFs and neovascularization, long-term hypoxic conditions in wound environments may preclude the healing cascade. In burn injuries, damaged vascular networks result in the loss of oxygen and nutrient supply to tissues, as well as the aberrant production of ROS from inflammatory neutrophils, which may delay skin repair. Therefore, oxygen delivery has also been exploited for skin regeneration (Table 2). Lu and coworkers prepared oxygen-releasing self-healing hydrogels by grafting gallic acid onto quaternized chitosan (QCS-GA), which was further crosslinked with oxidized hyaluronic acid (OHA) [20]. Oxygen-releasing microspheres were incorporated into these self-healing hydrogels. The potential of these oxygen-releasing hydrogels was studied in vitro and in vivo in a mouse burn injury model for up to 15 days under hypoxic conditions. The oxygen release was sustained for up to 10 days (O_2_ > 5%), which promoted the formation and maturation of blood vessels, suppressed the production of ROS, and induced the migration and tube formation of human umbilical vein endothelial cells (HUVECs). Moreover, these hydrogels increased collagen deposition as well as macrophage polarization from M1 to M2 phenotypes [20].

**Figure 3:**
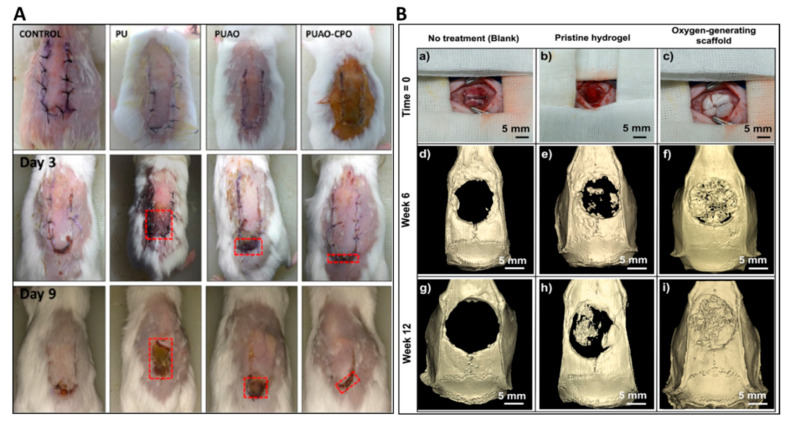
Effect of oxygen delivery on in vivo tissue regeneration of implanted scaffolds. (**A**) Elevated skin wound healing in the presence of PUAO and CPO-loaded PUAO as compared to control and bare PU groups. (**B**) Effect of oxygen-generating microparticles (OGMs) on the regeneration of critical-sized bone defects: (**a**,**d**,**g**) control group with minimal bone formation, (**b**,**e**,**h**) pristine hydrogel with a little regeneration, (**c**,**f**,**i**) OGM-mediated elevated bone regeneration by week 12. The figures were adopted from ref [21,48] with permission from Elsevier, Limited.

Since peroxide-based oxygen-producing biomaterials produce free radicals as reaction intermediates, the accumulation of these free radicals may increase oxidative stress, thus reducing cell viability. To cope with this limitation, natural antioxidants, such as catalase, are incorporated into oxygen-producing biomaterials to attenuate the production of oxygen free radicals. However, the incorporation of enzymes may further complicate the scaffold design; the incorporated enzymes may also lose their stability. Sheikh et al. designed oxygen-releasing biomaterials with antioxidative properties. Briefly, antioxidative polyurethane (PUAO) was employed to deliver CaO_2_ (PUAO-CPO). The polymer can attenuate free radicals owing to its antioxidative properties. The developed PUAO-CPO scaffolds suppressed ROS production and elevated cell survival under hypoxic conditions. The implantation of these scaffolds in an ischemic skin flap model diminished tissue necrosis for up to 9 days, as shown in Figure 3A [21].

Similarly, Leipzig and coworkers synthesized fluorinated methacrylamide chitosan (MACF)-based hydrogels by conjugating PFCs with methacrylamide chitosan via Schiff base nucleophilic substitution. Oxygen capture and release was improved with an increase in the PFC content for up to 5 days. Due to the high capacity of PFCs to absorb oxygen, MACF hydrogels absorbed oxygen for 2–6 h and afforded its continuous release for up to 120 h in vitro. The incorporation of PFC chains with a fluorinated aromatic group considerably enhanced oxygen uptake and extended release compared with linear PFC chains with the same numbers of fluorine molecules. In vitro culture of fibroblasts showed considerable growth, thereby indicating that an appropriate oxygen uptake and release may be instrumental both for in vitro cell culture and survival. Since *P*O_2_ or tension in normal subcutaneous tissues is 30–40 mmHg, dropping to 30 mmHg in acute dermal wounds and <5 mmHg in chronic diabetic ulcer patients, the application of these oxygen-producing antioxidative hydrogels may have great promise to provide oxygen in vivo at the wound site. While MACF-based hydrogels showed promising potential in alleviating hypoxia and furnishing oxygen in vitro, they lacked multiple functionalities in addition to being costly [22]. Therefore, multifunctional biomaterials manifesting anti-inflammatory, antibacterial, and efficient drug delivery functions are promising options, especially for chronic wound healing applications.

Gao and coworkers designed multifunctional, ROS-scavenging, O_2_-producing, and nitric oxide (NO)-generating hydrogels by exploiting hyperbranched poly-L-lysine (HBPL)-modified manganese dioxide (MnO_2_) nanosheets and pravastatin sodium for ROS-mediated oxygen production and NO generation. Hydrophilic poly(PEGMA-*co*-GMA-*co*-AAm) (PPGA) polymers were synthesized by the radical polymerization of poly(ethylene glycol) methyl ether methacrylate (PEGMA), glycidyl methacrylate (GMA), and acrylamide (AAm) [23]. The developed hydrogels suppressed bacterial quorum sensing (QS), presumably due to the antibacterial effect of HBPL as well as their ability to stabilize MnO_2_ nanosheets. MnO_2_ nanosheets enabled the production of O_2_ from ROS and NO due to the loading of pravastatin sodium before crosslinking. Accordingly, HBPL/MnO_2_ composites remarkably reduced oxidative stress by suppressing the generation of H_2_O_2_ and hydroxide radicals (OH). Moreover, these hydrogels reduced the production of ROS as well as pro-inflammatory factors, including interleukin-1-β (IL-1β), chemokine (C-X-C motif) ligand 1 (CXCL-1), tumor necrosis factor-α (TNF-α), and IL-6, while they increased the production of anti-inflammatory factor markers, such as transforming growth factor-beta (TGF-β) and IL-4 in a methicillin-resistant Staphylococcus aureus (MRSA)-infected diabetic wound model.

Unlike conventional oxygen delivery methods, such as CaO_2_, PFCs, and nanozymes, Centeno-Cerdas et al. exploited genetically modified microalgae as an oxygen source and fabricated photosynthetic sutures. Genetically modified microalgae containing recombinant GFs, including VEGF, platelet-derived growth factor-BB (PDGF-BB), and stromal cell-derived factor-1-alpha (SDF-1α) was employed. The natural mechanism of photosynthesis in these microalgae afforded continuous oxygen release for up to 14 days in vitro. While this modification approach may help circumvent the limitations of conventional OCs, such as poor loading and short-term release of oxygen, it requires light penetration, and thus depends on the localization of the particular type of tissue [24].

Compared to normal wound healing, the repair and regeneration of diabetic wounds is complicated, as they are often accompanied by poor neovascularization, an overproduction of ROS, and hypoxic microenvironments in hyperglycemic conditions. This necessitates the development of multifunctional wound dressings with several tissue regenerative functionalities in a single application. To circumvent these limitations, Xiong et al. fabricated a HA-based composite hydrogel containing MnO_2_ (MnO_2_/ePL) nanozymes, which alleviated an overproduction of H_2_O_2_. Hydrazide-modified HA and aldehyde-modified HA were reacted to form a Schiff base between the hydrazide moieties and aldehyde moieties. In addition, M2 macrophage-derived exosomes (Exos) and fibroblast growth factor-2 (FGF-2) were also incorporated into these hydrogels to induce macrophage polarization from M1 to M2 phenotypes. These oxygen-producing hydrogels promoted angiogenesis and epithelialization. Overall, the HA@MnO_2_/FGF-2/Exos based hydrogel system promoted collagen deposition, granulation tissue formation, and angiogenesis, thereby suggesting a potential therapeutic strategy for diabetic wound healing [25].

**Table 2:** Oxygenated scaffolds for skin regeneration.

Material Type	Oxygen Carrier	Regenerative Effects	Ref.
Gallic-acid-grafted quaternized chitosan	O_2_-releasing microsphere	O_2_ release was sustained for 10 d Blood vessel formation was increased Production of ROS decreased Tube formation of HUVECs improved Collagen deposition increased Macrophage polarization from M1 to M2 phenotypes	[20]
Fluorinated methacrylamide chitosan	Perfluorocarbon (PFC)	O_2_ released for up to 5 d Survival and growth of fibroblasts increased Hypoxia decreased	[22]
Poly(PEGMA-Co-GMA-Co-AAm) (PPGA)	Poly(L-lysine) modified manganese oxide (MnO_2_)	Bacterial quorum sensing decreased O_2_ production from ROS and NO increased Oxidative stress decreased Pro-inflammatory factors decreased Anti-inflammatory factors increased	[23]
Hyaluronic acid (HA)	Manganese oxide/poly(L-lysine) nanozyme, M2-derived exosomes, and fibroblasts growth factor-2 (FGF-2)	Collagen deposition and granulation increased Angiogenesis increased Antibacterial properties increased Diabetic wound healing increased	[25]

#### 2.1.2. Heart

Myocardial infarction (MI) remains the foremost clinical challenge causing enormous deaths worldwide. The major factors resulting in MI include coronary artery blockage, which leads to the poor diffusion of oxygen and transport of nutrients to cardiomyocytes [26]. This is further exacerbated by an overproduction of ROS under hypoxia and subsequent cell death, which may lead to chronic heart failure [18]. Oxygen delivery systems have also been exploited to promote the regeneration of infarcted myocardial tissues (Table 3). Gao and coworkers designed ROS-scavenging and O_2_-producing injectable hydrogels for the regeneration of infarcted myocardia. Hyperbranched polymers containing thioketal groups (HBPAK) were copolymerized with methacrylated hyaluronic acid (HA-MA); catalase enzymes were encapsulated into the scaffolds by ultraviolet (UV) irradiation. HBPAK was synthesized by Michael addition between polyethylene glycol diacrylate and thioketal diethyl amine. The catalase enabled the decomposition of H_2_O_2_ into H_2_O and O_2_, which promoted macrophage polarization from M1 to M2 phenotypes, induced angiogenesis, and suppressed cell apoptosis [27]. In vivo results in an MI model revealed the ability of the hydrogels to eliminate excessive oxygen-related free radicals, alleviate hypoxia conditions, reduce inflammatory TNF-α level in serum, inhibit cell apoptosis, and increase the M2/M1 macrophage ratio. In addition, these oxygen-producing hydrogels showed significant improvements in heart function compared to that of the untreated groups.

Several approaches have been adopted to overcome hypoxia and suppress ROS production for infarcted myocardium regeneration, including the delivery of OCs, as well as the direct injection of supersaturated oxygen. Sheikh et al. fabricated PU-based cardiac patches and incorporated CaO_2_ as OCs. These patches provided the continuous release of O_2_ for up to 10 days in vitro, promoted the survival and growth of ADSCs, and induced the formation of tubular networks. Implantation of these patches in an MI model improved cardiac function normalization and induced blood vessel formation and cardiac remodeling, while suppressing fibrosis and oxidative stress [28].

Similarly, Mandal et al. prepared CaO_2_-loaded MPs and incorporated them into PLGA. The developed MPs released O_2_ for up to 24 h in a sustained manner and promoted the metabolic functions of neonatal cardiac myocytes in hypoxic conditions, restored the reduced energy of hypoxic cells, and improved cardiac contractility. These oxygen-releasing hydrogels also alleviated MI-related cardiac stress in rabbit heart tissues ex vivo [29].

#### 2.1.3. Nerves

Injuries of the central nervous system (CNS) and peripheral nervous system (PNS) pose huge discomfort worldwide and necessitate nerve regeneration. Different types of methods have been adopted to promote nerve repair, including cell transplantation, drug/GFs delivery, and implantation of artificial nerve guidance conduits (NGCs). NGCs leverage a conducive environment for nerve repair by promoting the regeneration of remyelinated axons and impeding the infiltration of inflammatory cell types, such as astroglia and microglia [30]. For nerve repair, Schwann cells (SCs) play a pivotal role in the myelination of peripheral nerves, and also leverage neurotrophins and ECM components for axonal outgrowth and elongation. However, the transplanted SCs lack optimum functionalities due to poor vascularization and reduced oxygen diffusion during nerve repair. Therefore, oxygen delivery to SCs prior to vascularization of the graft may hold great promise for the survival and growth of SCs. To circumvent hypoxia in nerve grafts and afford a sustained release of oxygen for an extended period of time, Ma et al. fabricated core/shell type fibers by co-axial electrospinning [24]. Perfluorotributylamine (PFTBA) was used as an oxygen carrier and was incorporated into the core of the fibers; the shell was composed of PCL fibers. PFTBA was chosen as an oxygen carrier owing to its good biocompatibility and lower production of ROS compared to peroxides.

SCs exhibited high viability and growth, as well as the production of more angiogenic and neurotrophic GFs, such as VEGF, nerve growth factor (NGF), and brain-derived neurotrophic factor (BDNF), in PFTBA-containing fibers than the control group, presumably due to the sustained release of oxygen. The in vivo implantation of PFTBA-loaded conduits promoted the viability of SCs for up to 14 days and afforded significant axonal regeneration and remyelination [31]. The elevated viability and growth of SCs was observed in the PFTBA hydrogel group owing to the ability of PFTBA to supply oxygen to SCs, which can mitigate cellular hypoxia during the initial stages of implantation. Therefore, GFP^+^ SCs were significantly higher in the PFTBA hydrogel group as compared to the control group, even after 28 days of implantation. Thus, enhanced nerve regeneration was attributed to enhanced SCs’ survival during the initial stages, which promoted VEGF release from SCs and further promoted the vascularization of nerve scaffolds. In a recent study by the same group, PFTBA and VEGF were co-loaded into the core part of the fibers, which overcame short-term oxygen delivery issues in the nerve conduits. The concurrent delivery of VEGF supported microvessel formation after exhaustion of the PFTBA-mediated O_2_ release (ca. 140 h). The developed PFTBA–VEGF-based conduits significantly promoted the survival of SCs under hypoxia and induced the migration and tubule-like network formation of HUVECs in vitro. The PFTBA–VEGF conduits were transplanted into a 15 mm long sciatic nerve defect rat model for up to 2 weeks. Conduits loaded with PFTBA and VEGF remarkably improved the survival of SCs, axonal regeneration, and sciatic nerve index (SCI). The continuous release of VEGF for up to 190 h enabled the higher survivability of SCs in the dual-cue loaded conduits compared with that of the single-cue loaded groups [32].

We have observed the significant effect of VEGF-binding peptides or VEGF along with other bioactive cues, such as SDF-1α and NGF, on the survivability of SCs [30,33]. Consequently, different types of bioactive cues can be leveraged along with oxygen-producing biomaterials to improve tissue repair. It is anticipated that oxygen can increase neovascularization, while the bioactive cues can improve the mobilization and recruitment of stem/progenitor cells, as well as their differentiation into targeted lineages.

Similarly, Zhu et al. loaded PFTBA into collagen and chitosan-based NGCs. Olfactory ensheathing cells (OECs) were seeded into the conduits. The NGCs were fabricated by using a customized-designed mold into which the PFTBA–OECs containing fibrin gel were injected. The PFTBA improved the survival of OECs in vivo, which was attributable to the enhanced oxygen release at the earlier stages of cell transplantation. Thereby, the NGCs also promoted axonal regeneration, nerve regeneration, and vascularization [34].

Stroke is another complication, which leads to multiple disorders and even deaths, presumably owing to ischemia in the cerebral region. The blockage of a cerebral artery may further aggravate hypoxia and cause neuronal death. Wang et al. developed nanophotosynthesis therapy (NPT) by employing core/shell type neodymium (Nd3^+^)-doped upconversion NPs (UCNPs), which enabled the conversion of near-infrared light (NIR) into visible light; the latter was absorbed by the *S. elongatus* cyanobacteria. When evaluated in a middle cerebral occlusion (MCAO) model in mice, these UCNPs afforded O_2_ production and carbon dioxide (CO_2_) removal, which promoted the survival of neurons, induced the formation of neovessels, and reduced infarct volume, along with significant behavioral improvement [35].

#### 2.1.4. Pancreas

Type 1 diabetes (T1D) remains the most destructive autoimmune disorder rendering damage to pancreas beta cells, which ultimately leads to hyperglycemia. While insulin and immunosuppressants are used to treat T1D, these modalities underperform in chronic situations, which may cause several complications, such as neuropathy, nephropathy, and retinopathy. Transplantation of islets is the most effective strategy to deliver pancreatic beta cells for the continuous production of insulin. Nevertheless, pancreatic beta cells are metabolically more active and require a continuous supply of oxygen and nutrients. In addition, prolonged inflammatory response may limit graft survival and hamper the full utilization of islets. The isolated pancreatic beta cells undergo apoptosis due to hypoxic conditions at the transplantation site. Therefore, approaches to deliver a continuous supply of oxygen during the initial 5–10 days of transplantation may be beneficial to improve the survival and retention of islets partly via improved neovascularization.

McQuilling et al. used sodium percarbonate and CaO_2_ to enhance the survival of islets. SPO was used with or without silicon (Si) films, while CaO_2_ was incorporated into alginate microbeads. These materials afforded oxygen release for up to 7 days. However, further in-depth studies are needed to clearly understand oxygen release kinetics and their effect on cell survival and functioning [36].

Pedraza et al. leveraged CaO_2_ to improve cell survival under hypoxic conditions. CaO_2_ was encapsulated into PDMS; the hydrophobicity of PDMS allowed prolonged O_2_ release for up to 6 weeks (concentration of the released O_2_: 0.06 mM per day). Unlike traditional approaches, which often require catalase for the release of O_2_, this approach without catalase can suppress the production of hydroxyl radicals (OH). Co-culture of MIN6 cell line and rat pancreatic islets along with the oxygen-producing scaffolds exhibited higher cell survival as well as increased metabolic functions alongside reduced hypoxia-induced cell dysfunction and apoptosis. Nonetheless, owing to the suppression of hypoxia-mediating pathways, the production of angiogenic GFs can be limited, which requires further careful investigations [37].

Montazeri et al. fabricated core/shell type MPs; core and shell layers were composed of polyvinylpyrrolidone/H_2_O_2_ (PVP/H_2_O_2_) and PLGA, respectively. For the sustained release of oxygen, tetrameric groups of catalase were covalently conjugated with the carboxylic groups (-COOH) of PLGA in the shell layers. These MPs were dispersed into fibrin- and heparin/VEGF-based scaffolds and were used to deliver islets. The MP-containing scaffolds significantly reduced hypoxia for up to 7 days and improved the survival of the MIN6 β cell line and pancreatic islets in vitro. Co-transplantation of MP- and collagen-based scaffolds containing rat islets and heparin/VEGF significantly improved graft revascularization while reduced euglycemic time in the omenta of T1D model mice. These results show that the concurrent release of oxygen and angiogenic factors may be a viable approach to improve cell survival at earlier stages and warrant further studies by reducing the number of transplanted islets and insulin dependency [38].

While the abovementioned types of scaffolds can promote the survival of islets, as well as improve cell viability before the vascularization of grafts, the limited diffusion of oxygen and hypoxic microenvironment at the transplantation site may compromise the therapeutic benefits. To circumvent these limitations, in situ oxygenation through an O_2_ device has been proposed to improve O_2_ concentration surrounding the grafts [39]. While effective, these approaches require a complicated design of an O_2_ device as well as patients’ compliance to O_2_. To improve this design, Wang et al. exploited an insect-tracheal-system-inspired O_2_ release system, which was composed of an internal porous skeleton composed of superhydrophobic poly(vinylidene fluoride-co-hexafluoropropylene) (PVDF-HFP) and an external hydrophilic polydopamine (PDA) coating for the diffusion of O_2_ from the transplantation sites. This device afforded 1.8-fold higher O_2_ release and significantly promoted cell survival under hypoxic conditions. Similarly, in the in vivo implantation, these scaffolds improved the viability and survival of islets for up to 6 months [40].

**Table 3:** Oxygenated scaffolds for soft tissue regeneration.

Polymer	Oxygen Carrier	Findings *	Ref
PDMS	CaO_2_	O_2_ release for up to 6 weeks Survival and metabolic function of islets and MIN6 cells increased Cell apoptosis decreased	[6]
Polyurethane (PU)-based cardiac patches	CaO_2_	O_2_ released for 10 days Survival and growth of ADSCs increased Cardiac function and neovascularization improved Fibrosis and oxidative stress decreased	[28]
PLGA	CaO_2_-loaded particles	O_2_ released for up to 24 h Metabolic function of myocytes increased Cardiac stress decreased ex vivo	[29]
PCL-based core/shell type fibers	Perfluorotributylamine (PFTBA)	Viability and growth of SCs increased Production of NGF, BDNF, and VEGF increased Viability of SCs increased for up to 14 d in vivo Axonal regeneration increased	[31]
PCL-based core/shell fibers	PFTBA-VEGF co-delivery	Release of O_2_ for up to 190 h Co-delivery of VEGF and PFTBA increased the growth of microvessels Survival of SCs increased under hypoxia Survival of SCs also increased in a sciatic nerve defect model Sciatic nerve index and axonal regeneration improved The VEGF/PFTBA co-loaded group showed higher survival of SCs than that of the single cue loaded group	[32]
Chitosan- and collagen-based nerve guidance conduits	PFTBA	Survival of olfactory ensheathing cells increased Axonal regeneration, nerve regeneration, and vascularization increased	[34]
Fibrin and heparin/VEGF-based scaffolds	PVP/H_2_O_2_ and PLGA-based core/shell type NPs	Hypoxia reduced for up to 7d Cell survival increased In vivo vascularization in a diabetic model improved	[38]

* Nerve growth factor (NGF); brain-derived neurotrophic factor (BDNF); vascular endothelial growth factor (VEGF); Schwann cells (SCs); adipose-derived stem cells (ADSCs).

#### 2.1.5. Muscle

Muscle tissue can be damaged or degenerated due to multiple diseases, traumatic injuries, or tumor progressions which can potentially result in muscle loss. Ischemic conditions especially limit recovery, which necessitates the deployment of regenerative approaches. Therefore, OGBs for muscle tissue are essentially required. However, only a few studies have employed OGBs for the oxygenation of muscle tissues, a field which necessitates considerable attention [41]. HBO and peroxides, including CaO_2_ and Na_2_O_2_, have been exploited for the oxygenation of muscle tissues, and encouraging results have been obtained.

Ward et al. studied the effect of Na_2_O_2_ on hypoxic and ischemic skeletal muscle cells, and a higher cell survival rate was observed in the presence of Na_2_O_2_ [42]. Physiologically compatible ranges of Na_2_O_2_ improved the maintenance of contractility and attenuated the accumulation of HIF1α, depletion of intramuscular glycogen, and oxidative stress (lipid peroxidation) that occurred following ~30 min of hypoxia in muscle tissues. These findings demonstrated considerable potential for oxygen delivery for the homeostasis of skeletal muscle tissues. Similarly, Seifu et al. exploited fluorine-based zeolite particles as OCs and assessed the effect of oxygen carriers on in vitro cell survival and proliferation. Zeolites were fluorinated with perfluorodecyltriethoxysilane (PFTES) and embedded into 3D-printed PU scaffolds. The scaffolds containing fluorinated zeolites provided an elevated oxygen concentration and promoted the proliferation of human coronary artery smooth muscle cells and increased cell infiltration compared to the control group [43].

### 2.2. Oxygenated Scaffolds for Bone Regeneration

#### Bone

In addition to the regeneration of soft tissues, the repair and regeneration of load-bearing hard tissues, including bone, also remains a persisting challenge in regenerative medicine and TE. Bioactive ceramics as well as inorganic–organic composites are often exploited for bone TE [44]. In addition, vasculogenic and osteogenic GFs and the related biologics are incorporated into scaffolds to simultaneously promote angiogenesis and osteogenesis for timely bone repair [45,46]. During the initial 4–6 weeks of the implantation of scaffolds for bone TE, vascularization may provide the diffusion and transport of oxygen and nutrients for bone repair. Inefficient neovascularization may lead to oxidative stress, hypoxia, and cell apoptosis [18]. Therefore, oxygen delivery during this phase is essential to maintain tissue variability and metabolic functions (Table 4).

Farris et al. developed polyvinyl alcohol (PVA) and PLGA-based core/shell type microtanks (µtanks). The core was comprised of PVA to reduce the oxygen release, while the shell was made of PLGA to retard the degradation of the scaffold. These µtanks were then loaded into 3D-printed PCL scaffolds. The 3D-printed scaffolds were loaded with 100% O_2_ gas. These oxygen-producing scaffolds were used to enhance the survival of human adipose derived stromal/stem cells (hADSCs). O_2_ was released for up to 8 h in vitro. Scaffolds were transplanted into a subcutaneous model and a calvarial defect model. Interestingly, the initial short-term release of O_2_ improved ECM deposition and induced the greater production of osteogenic proteins. Scaffolds were evaluated for up to 8 weeks and were shown to remain structurally stable. These results showed the beneficial effect of the initial O_2_ release for the upregulation of genes, which may also have implications for enhancing the biomineralization for long-term implantation [47].

Suvarnapathaki et al. fabricated organic–inorganic hybrid NPs based on PCL and CaO_2_, which were dispersed in methacrylated gelatin (GelMA). These scaffolds showed the continuous release of oxygen for up to 2 weeks in vitro and reduced the direct contact of CaO_2_ with the surrounding microenvironment. Consequently, the viability and growth of preosteoblasts was remarkably increased. Implantation of these grafts in a calvarial defect model promoted bone regeneration, thereby manifesting significantly more new bone tissues and vascularization than the control group (Figure 3B) [48].

It is well-established that MSCs are the most widely used cells in transplantation studies owing to their potential to differentiate into multiple cell lineages, including osteoblasts and chondrocytes. For bone regeneration, the osteogenic capability of scaffolds has been further improved with the incorporation of bone morphogenetic proteins (BMP) by using recombinant engineering technology. However, cell survival was still limited due to hypoxia. Kimelman-Bleich et al. loaded BMP-2 and PFTBA into fibrin-based hydrogels along with MSCs and assessed the performance of scaffolds in a radial bone defect model and a spinal fusion model in mice in vivo. While BMP-2 improved cellular function via autocrine fashion, PFTBA promoted the viability of MSCs which altogether promoted bone regeneration, osteocalcin formation, bone quality, and bone volume in both models. However, further mechanistic studies yet remain to be performed [49].

While these approaches markedly elevated bone tissue regeneration by enhancing cell survival under hypoxic conditions, they lacked antibacterial functions which may also limit tissue regeneration, thereby compromising the integration of the implants. To circumvent these limitations, Huang et al. fabricated hybrid scaffolds consisting of quaternized methacrylated chitosan and HAp@PDA-F (hydroxyapeptite (HAp), polydopamine (PDA), and PFC NPs). These scaffolds provided oxygen release for up to 1 week and promoted the survival, growth, and osteogenic differentiation of BM-MSCs. Intriguingly, these scaffolds also exhibited antibacterial effects against *S. aureus* and *E. coli*, which were ascribed to the presence of fluorine. These scaffolds were further implanted in calvarial defect models in rats for up to 4 and 8 weeks, which showed improved bone growth and bone mineral density (BMD) [50].

**Table 4:** Oxygenated scaffolds for hard tissue regeneration and miscellaneous.

Scaffold Material	Oxygen Carrier	Regenerative Effects	Ref.
Polypropylene sulfide (PPS)-based microspheres	Curcumin	ROS-responsive release of curcumin Cell apoptosis decreased ROS scavenging in a hind-limb ischemia model	[45]
PVA/PLGA-based core/shell fibers	100% O_2_	ECM deposition and osteogenic protein expression increased in subcutaneous and calvarial defect models	[47]
Methacrylated gelatin	PCL/CaO_2_ NPs	O_2_ released for 2 wks Viability and growth of preosteoblasts increased Oxygenated scaffolds showed higher bone formation in a rat calvarial defect model	[48]
Fibrin-based hydrogels	Bone morphogenetic protein-2 (BMP-2) and PFTBA	PFTBA promoted the survival of MSCs as well as other parameters for bone regeneration BMP-2 improved cellular functions	[49]
Quaternized chitosan, hydroxyapatite, and polydopamine-based NPs	Perfluorocarbon NPs	O_2_ released for up to 1 wk Survival, growth, and osteogenic ability of bone marrow mesenchymal stem cells (BM-MSCs) increased Antimicrobial effect by fluorine	[50]
Indocyanine green (ICG)-conjugated hyaluronic acid NPs	MnO_2_ NPs	In situ O_2_ production increased H_2_O_2_ decreased Tumor hypoxia decreased Tumor growth decreased Improved efficiency of PDT	[51]

## 3. Oxygen Delivery to Mitigate Tumor Hypoxia

As compared to normal tissues, excessive cell growth, neovascularization, and a degenerated tumor microenvironment lead to elevated oxygen consumption and nutrient requirements [52]. Owing to this, cancer cells often exhibit hypoxia, which limits the efficacy of anticancer treatments, including photodynamic therapy (PDT), radiotherapy, and chemotherapy. In addition, tumor hypoxia may cause multidrug resistance and cancer metastasis. Therefore, manipulation of hypoxia in tumor tissues is of considerable significance for cancer therapy. Oxygen-generating biomaterials have also been used to deliver oxygen in tumor models and have been shown to improve the efficacy of cancer therapy [53]. Gao et al. used MnO_2_ NPs to improve the efficacy of PDT. Indocyanine green (ICG) was conjugated to HA NPs for the absorption of NIR light, while MnO_2_ NPs were incorporated to induce the production of oxygen to decrease oxygen starvation. The hybrid NPs were internalized by the tumor cells; hyaluronidase enzyme degraded the HA and released MnO_2_. The released MnO_2_ afforded in situ production of O_2_, thereby decreasing an excessive production of H_2_O_2_. These hybrid NPs suppressed tumor growth and increased the efficiency of the PDT [51].

Tumor cells often exhibit hypoxia, which reduces the efficacy of tumor-targeted therapies. Therefore, the ablation of tumor hypoxia exhibits crucial significance. Hypoxic cells produce more ROS; H_2_O_2_ is predominant among the oxygenated molecules. Secondly, tumor cells overexpress CD44 surface receptors, while normal cells (e.g., NIH 3T3 fibroblasts) express a low level of CD44 surface receptors. Therefore, OCs containing HA enhanced the survival of fibroblasts while reducing the growth of tumor cells [51].

Motealleh et al. have observed a significant effect of oxygen-producing biomaterials on the proliferation of NIH 3T3 fibroblasts over that of cancer cells. The proliferation of cancer cells was significantly decreased with oxygen production, which was ascribed to oxygen-mediated destabilization of HIF-1α [11]. However, it is pertinent to mention that toxic products, residual ROS, and salt decomposition products may also pose additional risks, including an overproliferation of cells owing to a burst release of O_2_. Similarly, PFCs can persist within tissues, which can induce immune responses and other risks. Consequently, it is imperative to carefully elucidate the safety of oxygen-producing biomaterials to avoid risks.

## 4. Redox Scavenging Biomaterials Mitigating Oxidative Stress

ROS generated by all aerobic organisms are considered intensively reactive molecules, which include free radicals, such as hydroxyl radicals OH), superoxides (O_2_^−^), and certain non-radical groups, including H_2_O_2_ and singlet oxygen (^1^O_2_). While these species can regulate cell signaling mechanisms (e.g., cell growth, cell proliferation, cell differentiation, etc.) through catalases, glutathione peroxidases, and superoxide dismutase, an imbalance may lead to an overproduction of ROS, as well as oxidative stress and cell apoptosis [54].

Augmented oxidative stress in cells and tissues produces ROS which generate and complicate inflammatory pathological conditions, including peripheral arterial disease (PAD) [55]. The possible mechanism of ROS generation includes the infiltration and recruitment of leukocytes, especially macrophages, in the inflamed tissue, which produce ROS. The ROS further enhance the expression of leukocyte adhesion molecules on the endothelium, thereby further exacerbating leukocyte infiltration and ROS production [56]. For instance, in the case of diabetes, hyperglycemia results in an elevated level of superoxides, such as peroxynitrite, H_2_O_2_, and hydroxyl free radicals (OH). Therefore, diabetic patients are more susceptible to PAD, which can even result in limb amputation. To overcome PAD and other inflammatory and oxidative complications, ROS-scavenging biomaterials have gained considerable interest in the research community. Poole et al. fabricated curcumin-encapsulated poly(propylene sulfide) (PPS)-based microspheres. Oxidative species increased the degradation of hydrophilic PPS and enabled ROS-mediated curcumin release in vitro and in vivo. The cytocompatible curcumin-loaded PPS microspheres also reduced cell death and scavenged ROS in activated macrophages in vitro as well as in a hind-limb ischemia model in mice [57].

Larranaga et al. fabricated polymeric capsules by using a layer-by-layer assembly method. Calcium carbonate (CaCO_3_) was used as a sacrificial template; catalase enzymes were loaded into NPs, while tannic acid (TA) was coated on the surface of the particles. The catalase reduced the production of H_2_O_2_, while the TA further reduced the production of H_2_O_2_ and hydroxyl radicals (OH). Moreover, these polymeric capsules also reduced the production of ROS and matrix metalloproteinase-3 (MMP-3) in an IL-1β induced nucleus pulposus model in vitro [58].

While we have comprehensively reviewed oxygen-producing biomaterials, including different types of OCs and natural and synthetic polymer-based scaffolds as OCs, as well as their applications for soft and hard tissue repair, there are also several limitations in this study. We have mainly discussed recent findings related to oxygen-producing biomaterials and mainly focused on the studies related to in vivo applications. Since previously different comprehensive reviews have summarized the details of OCs, as well as the mechanism of oxygen generation, we have briefly outlined these aspects [3,4,5,6,7]. As discussed in this review, oxygen-producing biomaterials may be equally beneficial for in vitro tissue fabrication, tissue regeneration in vivo, and lowering oxidative stress, ROS, and tumor hypoxia. While we have briefly outlined the application of these materials in the abovementioned disciplines, we have mainly focused on the in vivo applications.

## 5. Conclusions

**Figure 4:**
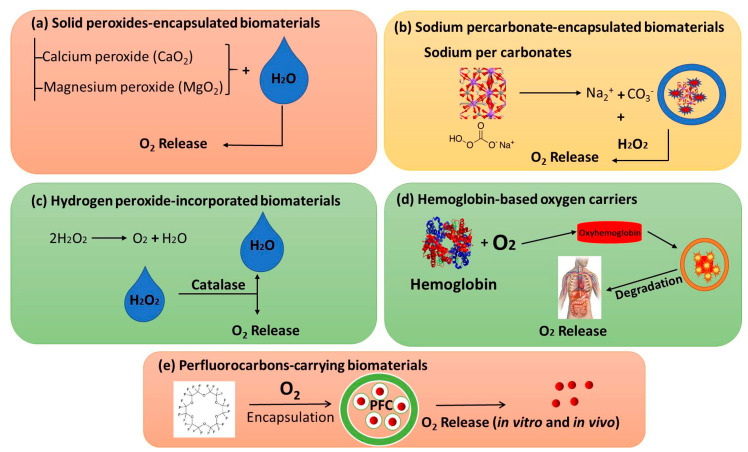
Mechanism for oxygen release from different types of oxygen-producing biomaterials.

In conclusion, different types of approaches have been pursued to enhance the oxygenation of implanted tissue constructs to enhance their in vitro and in vivo survival. These oxygen-producing biomaterials have been shown to provide sufficient oxygen diffusion for better cell survival in vitro and in vivo. Different types of biomaterial systems have been used for oxygen release for therapeutic benefits as summarized in Scheme 1 and Figure 1. These include hydrogels, NPs, nanofibers, electrospun scaffolds, 3D-printed scaffolds and so on. Similarly, different types of OCs, including molecular oxygen, Na_2_O_2_, CaO_2_, H_2_O_2_, and PFCs have been incorporated into oxygen-producing biomaterials (Figure 4). These oxygen generation/release systems have shown significant promise to improve cell survival in vitro and in vivo. For in vitro, OGBs have been shown to decrease hypoxia, oxidative stress, and pro-inflammatory markers and thus facilitate the formation of cell spheroids and organoids. Similarly, for the in vivo studies, oxygen-producing biomaterials have shown good potentials to improve the survival of transplanted pancreatic islets and hepatocytes. Moreover, the repair of different types of tissues, such as skin, heart, nerve, bone, and cartilage was also shown to be improved by the sustained oxygen generation/release by using different types of biomaterials.

Likewise, supramolecular dynamic biomaterials have been exploited as sustained and controlled drug delivery systems for a variety of therapeutics and nucleic acid drugs. Intriguingly, a few studies have also harnessed their potential as stimuli-responsive oxygen delivery systems [59,60]. Oxygen production systems have also shown considerable potential to improve cell survival, retention, and engraftment as well as facilitate graft survival in vitro and in vivo. Indeed, it has been deciphered that these benefits are due to an alleviation of oxidative stress and ROS as well as diminished hypoxia and inflammation modulation. Since tissue repair is a dynamic process, which requires a concerted effect of multiple signaling cues, including cell mobilization, cell recruitment, and cell differentiation, oxygen-producing biomaterials have also been used along with angiogenic/vasculogenic cues and neurotrophic factors to further expedite tissue repair. Similarly, oxygen-producing biomaterials have also shown promise for the oxygenation of tumor tissues to enhance the effectiveness of cancer therapy and surrogates for lowering oxidative stress for an effective tissue repair. Taken together, oxygen-producing biomaterials may offer an amenable platform to further enhance the functionality of biomaterials and scaffolds for regenerative medicine and TE applications.

## 6. Future Outlook

**Figure 5:**
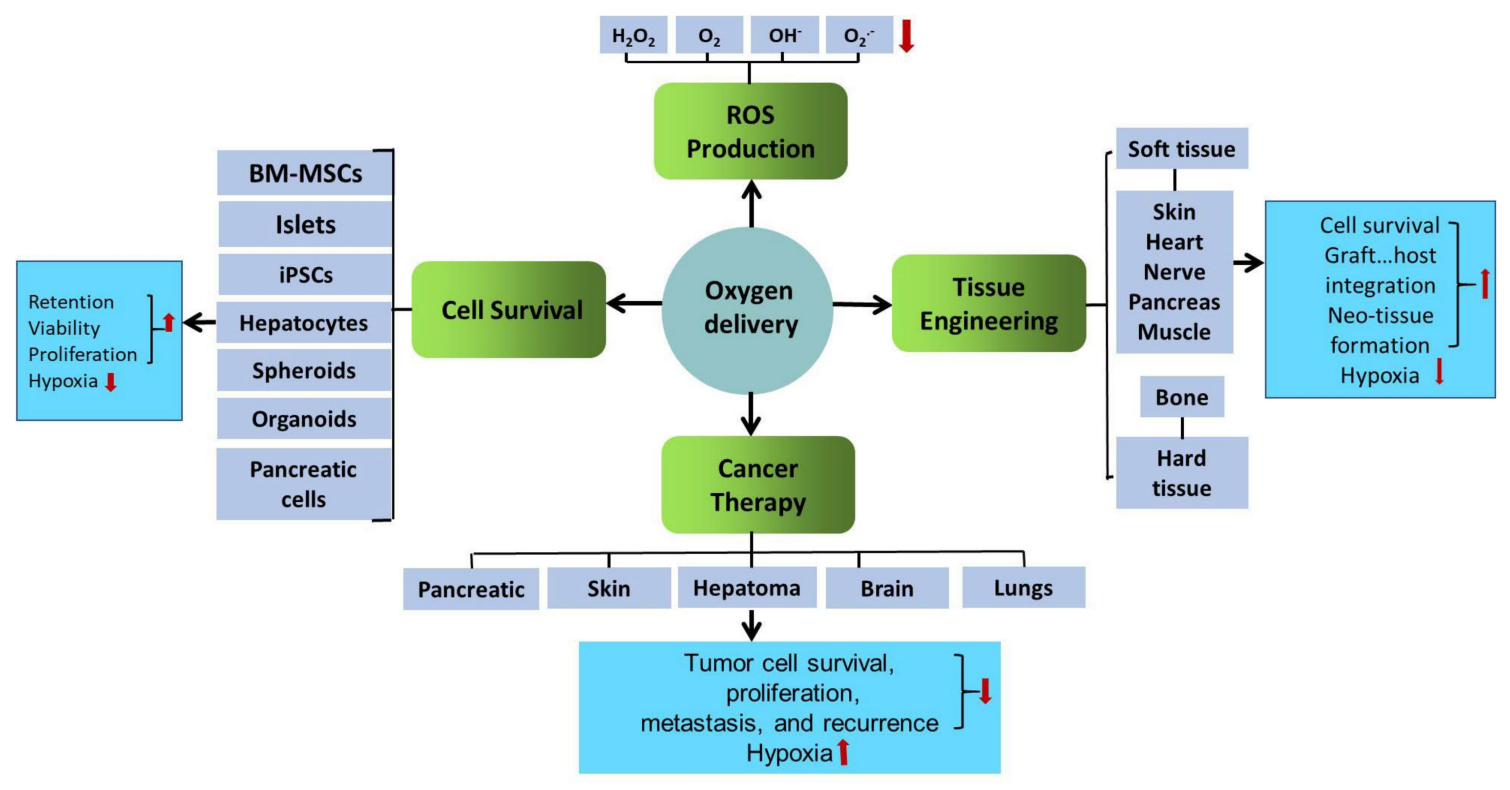
Schematic representation of the beneficial effects of oxygen delivery on in vitro and in vivo cell survival, tissue regeneration, and cancer therapy. The upward and downward arrows indicate an increasing and decreasing trend, respectively.

As mentioned in the preceding sections, oxygen-producing biomaterials have shown considerable potential for regenerative medicine and TE (Figure 5). However, there are several issues which still need to be carefully addressed. The foremost is the choice of an optimum oxygen carrier. Molecular oxygen is hard to retain in a delivery system and requires sophisticated material/experimental setup, while the direct use of RBCs as OCs is also impeded for clinical translation. Likewise, while H_2_O_2_ can cause oxygen production, it requires catalase enzymes; the latter may have a cytotoxic effect on healthy cells. On the other hand, the use of PFCs is further limited owing to their high volatility, as well as long-term retention. Both emulsion and conjugated systems have been exploited to improve the release of PFCs; conjugation systems produce good oxygen uptake and release in vitro and in vivo. Nevertheless, further optimization is still needed to achieve sustained oxygen production for good therapeutic benefits. Newer types of PFC-conjugated systems with different types of natural and synthetic polymers may offer an alternative paradigm for successful oxygen delivery for therapeutic gains in regenerative medicine and TE. Therefore, it is warranted that newer types of biomaterials should be developed as multifunctional OCs for regenerative medicine and TE applications.

It has previously been deciphered that hypoxic preconditioning of cells and hypoxia stabilization is beneficial for cell survival and tissue regeneration. Therefore, the distinct benefits and/or merits and demerits of each of these complementary approaches need to be elucidated in detail. Similarly, for successful tissue repair, there are several active players, such as soluble and tethered bioactive cues, GFs, and ECM components. Therefore, further strategies are needed to clearly dissect the role of oxygen delivery in context with the role of the abovementioned players. Furthermore, only a few studies have explored the synergistic effect of oxygen release and GFs, such as with VEGF [32]. Nonetheless, it has been revealed that the systematic delivery of oxygen and VEGF is beneficial for improving neovascularization. Therefore, further studies based on the concurrent delivery of oxygen along with GFs and other bioactive cues are needed.

The implication of OGBs can be hampered in certain pathological and ischemic conditions, such as MI or muscle injuries, where maximal or complete loss of blood supply may occur, thereby leading to the loss of oxygen supply even after the transplantation of OGBs/scaffolds. Therefore, novel scaffold designs and oxygen delivery approaches are required to overcome this barrier to achieve tissue oxygenation and the prevention of elevated cell death or tissue necrosis. Oxygen-producing biomaterials have been shown to improve oxygenation while reducing the elevated levels of ROS in infarcted myocardial tissues, along with beneficial effects. Sheikh et al. developed antioxidative PU-based scaffolds. CaO_2_ and ADSC-derived exosomes were incorporated into collagen fibers. These scaffolds attenuated cardiac remodeling, oxidative stress, and fibrosis, while increasing neovascularization [28]. Similarly, Gao and coworkers leveraged ROS-cleavable polythioketal-based hydrogels. For oxygen production, H_2_O_2_ and catalase were co-loaded into hydrogels. The developed scaffolds suppressed ROS production through ROS-mediated cleavage of polythioketal; H_2_O_2_ caused O_2_ release [26]_._ Consequently, oxygen-producing biomaterials may help overcome the loss of neovessels and promote oxygen diffusion through the sustained release of oxygen.

While the middle regions of implanted scaffolds express hypoxia, scaffolds exhibiting an average size of greater than 1 cm^3^ in particular do not receive a proper oxygen supply and can eventually experience ischemia, followed by necrosis [61,62]. Oxygen diffusion is nearly maintained at the distance of 100–200 µm from native capillaries in native tissues; thus, the transplantation of large-sized scaffolds containing metabolically active cells can lead to cellular hypoxia and subsequent cell death [21]. If the distance between the blood vessels and cells seeded within a tissue-engineered construct is more than 100–200 μm, cells will not survive, and this will ultimately lead to cell apoptosis and tissue necrosis [21]. Cells with high oxygen demand, such as cardiomyocytes, neurons, hepatocytes, and *β* cells within the pancreas are especially sensitive to oxygen deprivation and cease to function under hypoxic conditions. This can be mitigated by designing functional scaffolds, which may allow for the easy diffusion of produced oxygen. Indeed, micro/nanofabricated scaffolds containing microchannels have been shown to improve the diffusion of oxygen and the transport of nutrients. Similarly, hydrogels containing vascular networks have been shown to promote the anastomosis of scaffolds with the host-derived vasculature. Consequently, these types of strategies can also be leveraged along with oxygen-producing biomaterials to overcome hypoxia and oxygen diffusion problems for in vitro engineered thick-sized tissue constructs [47].

In silico and computational studies of biomaterials may provide an alternative paradigm for biomaterial evaluation and may also support in vitro, as well as in vivo, studies. Therefore, in silico and computational studies of biomaterials may also be performed for oxygen-producing biomaterials. It is noteworthy that in silico and computational studies are being widely performed for the precise design of biomaterials [63]. Last but not least, in addition to the types of materials or polymers employed to fabricate the scaffolds, their design also exhibits an essential role in post-implant success or failure; the scaffold porosity is considered an essential design element. Thus, an appropriate scaffold pore size may facilitate cell infiltration and migration after implantation [64,65].

## Data Availability

Not applicable.

## Abbreviations

**Full Name**	**Abbreviation**	**Full Name**	**Abbreviation**
Adipose-derived stem cells	ADSCs	Antioxidative polyurethane	PUAO
Acrylamide	AAm	Bone marrow	BM
Bone morphogenetic protein	BMP	Bone mineral density	BMD
Calcium peroxide	CaO_2_	Chemokine (C-X-C motif) ligand 1	CXCL-1
Central nervous system	CNS	Doxycycline	DOX
Exosomes	Exos	Extracellular matrix	ECM
Fibroblast growth factor-2	FGF-2	Fluorinated methacrylamide chitosan	MACF
Growth factors	GFs	Glycidyl methacrylate	GMA
Gallic-acid-grafted-quaternized chitosan	QCS-GA	Hyperbaric oxygen therapy	HBO
Human umbilical vein endothelial cells	HUVECs	Hyperbranched poly-L-lysine	HBPL
Hydroxyl radicals	OH	Hydrogen peroxide	H_2_O_2_
Holoparticles	HPs	Hypoxia-inducible factor 1 alpha	HIF-1α
Human red blood cells	hRBCs	Indocyanine green	ICG
Hyperbranched polymer containing thioketal groups	HBPAK	Interleukin-1 beta	IL-1β
Interleukin 4	IL-4	Manganese oxide	MnO_2_
Mesenchymal stem cells	MSCs	Microparticles	MPs
Myocardial infarction	MI	Methacrylated hyaluronic acid	HA-MA
Middle cerebral occlusion	MCAO	Methacrylated gelatin	GelMA
Nitric oxide	NO	Nanophotosynthetic therapy	NPT
Near-infrared light	NIR	N-isopropylacrylamide	NIPAAm
Nerve guidance conduits	NGCs	Nanomaterials	NMs
Oxygen partial pressure	*P*O_2_	Oxidized hyaluronic acid	OHA
Oxygen carriers	OCs	Oxygen-generating biomaterials	OGBs
Poly(L-lactide-co-glycolic acid)	PLGA	Polycaprolactone	PCL
Polyethylene glycol methyl ether methacrylate	PEGMA	PFC-based O_2_-carrying nanomaterials	PMOF
Polydimethylsiloxane	PDMS	Photodynamic therapy	PDT
Perfluorodecyltriethoxysilane	PFTES	Peripheral arterial disease	PAD
Perfluorocarbons	PFCs	Poly(lactide-co-ε-caprolactone)	PLCL
Poly(PEGMA-*co*-GMA-*co*-AAm)	PPGA	Platelet-derived growth factor-BB	PDGF-BB
Polyvinylpyrrolidone	PVP	Peripheral nervous system	PNS
Perfluorotributylamine	PFTBA	Poly(propylene sulfide)	PPS
PCL-based HPs loaded with PFO emulsion	PFO-HPs	Periodic mesoporous organosilica	PMO
Poly(vinylidene fluoride-co-hexafluoropropylene)	PVDF-HFP	Perfluoroctyl bromide	PFOB
Perfluorooctane	PFO	Quorum sensing	QS
Reactive oxygen species	ROS	Reversible addition–fragmentation chain reaction	RAFT
Sciatic nerve index	SCI	Sodium percarbonate	Na_2_H_3_CO_6_
Sodium alginate	Algl	Sodium carbonate	Na_2_CO_3_
Sodium peroxide	Na_2_O_2_	Stromal cell-derived factor-1 alpha	SDF-1α
Three-dimensional	3D	Tumor necrosis factor 1 alpha	TNF-α
Transforming growth factor-beta	TGF-β	Type 1 diabetes	T1D
Upconversion nanoparticles	UCNPs	Vascular endothelial growth factor	VEGF
